# Macrophage-B Cell Interactions in the Inverted Porcine Lymph Node and Their Response to Porcine Reproductive and Respiratory Syndrome Virus

**DOI:** 10.3389/fimmu.2019.00953

**Published:** 2019-05-03

**Authors:** Elise Bordet, Maxence Frétaud, Elisa Crisci, Edwige Bouguyon, Stéphane Rault, Jérémy Pezant, Alexis Pleau, Patricia Renson, Elisabetta Giuffra, Thibaut Larcher, Mickael Bourge, Olivier Bourry, Olivier Boulesteix, Christelle Langevin, Isabelle Schwartz-Cornil, Nicolas Bertho

**Affiliations:** ^1^VIM-INRA-Université Paris-Saclay, Domaine de Vilvert, Jouy-en-Josas, France; ^2^INRA, EMERG'IN- Plateforme d'Infectiologie Expérimentale IERP- Domaine de Vilvert, Jouy-en-Josas, France; ^3^UMR Génétique Animale et Biologie Intégrative, INRA, AgroParisTech, Université Paris-Saclay, Jouy-en-Josas, France; ^4^Department of Population Health and Pathobiology, College of Veterinary Medicine, North Carolina State University, Raleigh, NC, United States; ^5^INRA, UE1277, Plate-Forme d'Infectiologie Expérimentale, PFIE, Nouzilly, France; ^6^Anses, Laboratoire de Ploufragan-Plouzané-Niort, Unité Virologie et Immunologie Porcines, Zoopôle, BP53, Ploufragan, France; ^7^Université Bretagne Loire, Cité Internationale, Rennes, France; ^8^INRA, UMR 703 APEX, Oniris, Nantes, France; ^9^I2BC, Centre National de la Recherche Scientifique, Gif-sur-Yvette, France; ^10^BIOEPAR, INRA, Oniris, Nantes, France

**Keywords:** swine, lymph node, PRRSV, macrophage, B cell, centrocyte, BCL6

## Abstract

Swine lymph nodes (LN) present an inverted structure compared to mouse and human, with the afferent lymph diffusing from the center to the periphery. This structure, also observed in close and distant species such as dolphins, hippopotamus, rhinoceros, and elephants, is poorly described, nor are the LN macrophage populations and their relationship with B cell follicles. B cell maturation occurs mainly in LN B cell follicles with the help of LN macrophage populations endowed with different antigen delivery capacities. We identified three macrophage populations that we localized in the inverted LN spatial organization. This allowed us to ascribe porcine LN MΦ to their murine counterparts: subcapsular sinus MΦ, medullary cord MΦ and medullary sinus MΦ. We identified the different intra and extrafollicular stages of LN B cells maturation and explored the interaction of MΦ, drained antigen and follicular B cells. The porcine reproductive and respiratory syndrome virus (PRRSV) is a major porcine pathogen that infects tissue macrophages (MΦ). PRRSV is persistent in the secondary lymphoid tissues and induces a delay in neutralizing antibodies appearance. We observed PRRSV interaction with two LN MΦ populations, of which one interacts closely with centroblasts. We observed BCL6 up-regulation in centroblast upon PRRSV infection, leading to new hypothesis on PRRSV inhibition of B cell maturation. This seminal study of porcine LN will permit fruitful comparison with murine and human LN for a better understanding of normal and inverted LN development and functioning.

## Introduction

In most species, free antigens and DC migrating from the peripheral tissues enter the lymph node (LN) through the afferent lymphatic vessels into the external capsular sinus. Naïve lymphocytes enter the LN from the blood through the high endothelial venules (HEV) and then patrol the B and T cell areas. Upon antigen encounter in the context of the adequate antigen presenting cells, T and B lymphocytes get activated, mature and exit the LN through the medullary sinus and the efferent lymphatic vessel. Thus, most mammals, including mice and humans, possess LN with a centripetal lymphatic motion, i.e., afferent lymph enters the LN through the peripheral capsule and finds its way out toward the LN central hilum and the efferent lymph.

Conversely, in pigs (1), like in some species belonging to the *Laurasiatheria* superorder such as dolphins, hippopotamus (2), and rhinoceros (3), as well as in elephant (4), lymph presents a centrifuged motion. The porcine afferent lymphatic vessels enter the capsule at one site and penetrate deep into the area occupied by the B follicles and the T cells. Then they join the trabecular sinuses and filters into the subcapsular sinus from which efferent vessels originate (5). Naïve lymphocytes entered the LN through HEV as in other mammalian species, however, after having scanned the B and T cell areas, they exit directly in the blood through the same HEV (6).

In mouse, five populations of LN MΦ have been identified [for review (7, 8)]. The subcapsular sinus MΦ (SCS MΦ) (CD169^pos^/F4/80^neg^) transfer the antigens from the subcapsular space into the B cell follicle. SCS MΦ have been demonstrated as mandatory for mounting efficient cytotoxic (9) and humoral immune (10) responses. In the follicle, tangible body MΦ (TBM) scavenge the dead B lymphocytes whereas T cell zone MΦ (TZM) might do the same for T lymphocytes. The medullary cord MΦ (MCM) have a role in the plasma cells terminal maturation (11) and medullary sinus MΦ (MSM), situated at the exit of the LN would be involved in the final clearance of lymph borne particles.

Porcine reproductive and respiratory syndrome (PRRS) is a disease induced by the PRRS virus (PRRSV), a positive single stranded RNA virus from the *Arterivirade* family within the *Nidovirales* order (12). After oronasal transmission, PRRSV colonizes the respiratory tract and could play an immunomodulatory role delaying and weakening host responses, finally leading to virus persistence. Although anti-PRRSV antibodies are detected in the serum as early as one-week post-infection, the antibody serum titers to several viral proteins decline over time despite the continuous presence of the virus (13). Moreover, the emergence of neutralizing antibodies is strongly delayed, up to several months. Such delay has been proposed to be the main reason for PRRSV escape to the immune response [for review see (14)].

PRRSV strongly impacts the swine industry due to reproductive failures, reduced weight gain and predisposition to super-infections (15). The two main PRRSV cellular receptors are CD169/Sialoadhesin that allows the binding of the virus and CD163 which is essential for the release of the viral genome in the cytosol [for review see (16)]. PRRSV cellular targets are cells from the monocytic lineage, among them so far, only alveolar macrophages (MΦ) (17–19), pulmonary intravascular MΦ (20, 21) and CD163-positive tonsil macrophages (22) have been shown to be actually infected *in vivo*. We and others recently showed that *in vivo*, primary dendritic cells from lung (23) and tonsil (22) were not infected by the virus.

During the persistence phase, the virus is no longer detected in lung but could be isolated from LN up to 5 months post infection (24). Although virus are detected in secondary lymphoid tissues such as tonsils and lymph nodes, and at a lesser level spleen (25, 26), its target(s) in these tissues have not been precisely identified. One team studied the porcine LN MΦ for the sake of vaccine targeting to CD163 or CD169 (27, 28) but without making a distinction between different MΦ subpopulations. A study on the porcine respiratory tract draining LN upon PRRSV infection has been recently published (26). Using microdissection of follicular and interfollicular areas, the authors detected similar viral loads in both regions. The PRRSV ability to replicate in the respiratory draining LN for more than a month (25, 26) and the parallel delay in the appearance of neutralizing antibodies suggest that LN-MΦ infection by PRRSV might directly influence the B cell maturation process.

Herein, using whole LN imaging, flow cytometry analysis, cell sorting and RT-qPCR, we first described the localization and phenotype of three different MΦ subpopulations of the pig respiratory LN and assign each of them to their likely mouse counterpart. We described the different B cell maturation stages according to their expression of key transcription factors of B cell differentiation and their follicular localization. Subsequently, *in vivo* PRRSV infections were performed in order to study the susceptibility to infection of previously identified cells and to tentatively get information on how PRRSV infection may impacts the B cell maturation process.

## Materials and Methods

### *In vivo* Infections

Two different strains of the European originated PRRSV1 species were used: the PRSSV1.1 emergent Flanders13 (Fl13) strain (25) and the PRRSV1.3 highly pathogenic Lena strain (29). For *in vivo* experiments, PRRSV infections were performed at INRA PFIE (Nouzilly, France) for FL13 and ANSES (Ploufragan, France) for Lena infections. The animal experiments were authorized by the French Ministry for Research (authorization no.2015051418327338 and no.2015060113297443, respectively) and approved by the national ethics committee (authorizations no.09/07/13-1 and no.07/07/15-3). Ten-week-old Large White piglets were tested PRRSV free and inoculated intranasally at 5.10^5^ TCID50/animal or mock inoculated. For FL13, 3 pigs were used per group and euthanized 5 days post infection (dpi). For Lena, 4 pigs were used per group and euthanized at 10 dpi. Tracheobronchial LN were collected and processed as described above.

### Cell Isolation

Respiratory, tracheobronchial lymph nodes were collected from Large White conventionally bred sows from Guy Harang slaughterhouse (Houdan, France) and from the controlled UE-PAO-INRA (Nouzilly, France) herd. Tissues were minced and incubated with RPMI 1640 supplemented with 100 IU/ml penicillin, 100 mg/ml streptomycin, 250 ng/ml Fungizone® (Antibiotic-Antimycotic 100X ThermoFisher Scientific, Illkirch, France), 2 mM L-glutamine and 10% inactivated fetal calf serum (FCS, Invitrogen, Paisley, UK). Digestion were performed for 30 min at 37°C with 2 mg/ml collagenase D (Roche, Meylan, France), 1 mg/ml dispase (Invitrogen) and 0.1 mg/ml DNase I (Roche). Filtration on 40 μm cell strainers were performed and red blood cells were lysed using erythrolysis buffer (10 mM NaHCO3, 155 mM NH4Cl, and 10 mM EDTA). Cells were washed in PBS/EDTA and further processed fresh for flow cytometry staining and sorting as much as possible. Alternatively, LN cells were frozen in FCS + 10% dimethyl sulfoxide.

### Immunohistochemical Staining

Tracheobronchial LN were frozen in Tissue Teck (Sakura, Paris, France) and conserved at −80°C. Sections of 14 μm were obtained using a cryostat (Leica CM3050S, Nanterre, France) and deposed on Superfrost® glass slides (ThermoFisher scientific). Cryosections were fixed in methanol/acetone (1:1) at −20°C for 20 min. Fixed slides were saturated using PBS supplemented with 5% horse serum (HS) and 5% swine serum (SS) for 30 min at room temperature (RT). When biotinylated antibodies were used, a specific step of endogenous biotins saturation using Avidin/Biotin Blocking Kit (Invitrogen) was added. Primary and secondary antibodies (Table 1) were added at 4°C overnight or 30 min, respectively.

**Table 1:** Antibodies used for tracheobronchial lymph nodes staining for immunofluorescence and cell sorting.

**Antibody**	**Clone**	**Isotype**	**Species**	**Dilution**	**Supplier**
anti-CD169	1F1	IgG2a	mouse	pur	J Dominguez (INIA)
anti-CD21	B-ly4	IgG1	mouse	1/100	BD Pharmingen
anti-CD21-BV510	B-ly4	IgG1	mouse	1/50	BD Pharmingen
anti-CD8a	PT81B	IgG2b	mouse	1/100	MAb Center WSU
anti-CD163	2A10/11	IgG1	mouse	1/100	Biorad
anti-CD163-PE	2A10/11	IgG1	mouse	1/20	Biorad
anti-IgM	PG145A	IgM	mouse	1/150	MAb Center WSU
anti-CD172a/Sirpα	74-22-15a	IgG2b	mouse	1/250	MAb Center WSU
anti-CD2-Biot	RPA-2-10	IgG1	mouse	1/50	eBioscience
anti-Ki67-A555	B56	IgG1	mouse	1/20	BD Pharmingen

For beads draining experiments, tracheobronchial explants were injected at multiple sites in proximity of the target LN with 0.1 μm red fluorescent beads (FluoSpheres™ Carboxylate-Modified Microspheres, 0.1 μm, red fluorescent (580/605), 2% solids, ThermoFisher Scientific) diluted 1/4 in physiological serum. Explants were incubated 30 min à 37°C to allow the drainage of beads into the LN. The targeted LN was then sampled and frozen in Tissue Teck and processed as above for immunohistochemical staining, except for the fixation, in 4% PFA, 15 min at room temperature, in order to avoid bleaching of the beads' fluorescence by methanol, and the saturation and staining steps done in PBS, 5% HS, 5% SS, supplemented with 0.5% Triton in order to permeabilize the tissue upon PFA fixation.

Slides were analyzed using Zeiss AXIO Observer.Z1 microscope and Zen 2012 Software (Zeiss, Jena, Germany).

### Whole LN Staining and Clearing

Tracheobronchial LN was fixed by overnight incubation in paraformaldehyde 4% at 4°C. Immunohistochemical staining and clearing were performed following the iDISCO+ protocol (30). The sample was acquired on a light-sheet ultramicroscope (LaVision Biotec, Bielefeld, Germany) with a 2x objective. Whole LN and isolated follicles were acquired using 0.63X and 4X zoom factor, respectively. LN was reconstructed in 3D using Imaris 9.1 (Bitplane). The number of follicles was manually quantified.

### Flow Cytometry Analysis

Cells were stained in blocking solution, composed of PBS-EDTA (2 mM) supplemented with 5% HS and 5% SS. Staining were made in 4 steps, including PBS/EDTA with 2% FCS washing between each step: Uncoupled primary anti-CD169, anti-IgM and anti-CD172a antibodies were added to the blocking solution for 30 min on ice and then washed. Fluorescent, secondary, mouse isotype specific antibodies were then added, respectively anti-IgG2a-PE-Cy7, anti-IgM-Alexa647 and anti-IgG2b-APC-Cy7 for 20 min on ice and then washed. Then, to saturate the potential unbound IgG1-directed secondary antibodies sites, we incubated cells 30 min with isotype-control IgG1 (10 μg/ml) in blocking solution. Third the fluorochrom-coupled or biotinylated primary IgG1: anti-CD21 coupled to BV510, anti-CD163 coupled to PE and biotinylated anti-CD2 antibodies were added for 30 min on ice and then washed. Finally, streptavidin-coupled to Alexa700 was added for 20 min on ice and washed before resuspension in DAPI containing buffer for sorting (Table 1).

### Cell Sorting

LN cells were stained as for flow cytometry analysis and the different populations were sorted. Since LN cells are fragile the sorting was carried out on fresh cells to maximize cell yield and viability. Dead cells were excluded by DAPI staining (Sigma-Aldrich). Cells were sorted using a Moflo Astrios sorter (Beckman-Coulter, Paris, France) driven by summit 6.2. Puraflow 1X was used as sheath and run at a constant pressure of 25 PSI. Frequency of drop formation was 43 kHz. The instrument used a 100-μm nozzle. Temperature was kept at 4°C during whole sorting. The lasers used for excitation were solid state 405, 488, 561, and 640 nm. Optical filters used for collecting fluorescence were 448/59 nm (DAPI), 546/20 nm (BV510), 579/16 nm (PE), 671/30 nm (Alexa 647), 722/44 nm (Alexa 700), and 795/70 (PE-Cy7) and (APC-Cy7).

FlowJo software (version X.1.0, Tree Star, Ashland, OR, USA) was used for analysis.

### May-Grünwald-Giemsa Staining

Sorted cells were fixed and stained with May-Grünwald-Giemsa methods as previously described (31). Images were acquired and analyzed with a Pannoramic Scan II (3DHISTECH Ltd., Budapest, Hungary).

### RNA Extraction, Reverse Transcription, and Real-Time qPCR

Total RNAs from sorted cell populations were extracted using the Arcturus PicoPure RNA Isolation kit (ThermoFisher Scientific, St Aubin, France) according to the manufacturer's instructions. The Qiagen RNase-Free DNase Set (Courtaboeuf, France) was used to remove contaminating genomic DNA. Reverse transcription (RT) was made using Multiscribe reverse transcriptase (ThermoFisher Scientific) according to the manufacturer's instructions and qPCR carried out using the iTaq Universal SYBR Green Supermix (Biorad, Hercules, CA). Ribosomal protein S24 (RPS24) was chosen as reference gene as previously described in pig lung (31, 32). Primers used in this publication are reported in Supplementary Table 1. Using the porcine new born pig trachea epithelial cell line NPTR (33) we validated Topoisomerase IIA and Cyclin B2 as transcriptomic markers of cell proliferation, since their expression by RT-qPCR were respectively 30 and 17 times higher in exponential proliferation cultures compared with confluent NPTR cells. Conversely Ki67, which is a good proliferation marker at protein level, was invalidated for transcriptomic studies since its expression only varied by a factor of 2 between proliferating and confluent cells (Supplementary Figure 1a).

### Statistical Analysis

All data were analyzed using GraphPad Prism. The unpaired, non-parametric Mann-Whitney Statistical test was used.

## Results

### Macrophages Identification and Localization in the Tracheobronchial LN by CD169, CD163, and CD21 Microscopic Staining

**Figure 1:**
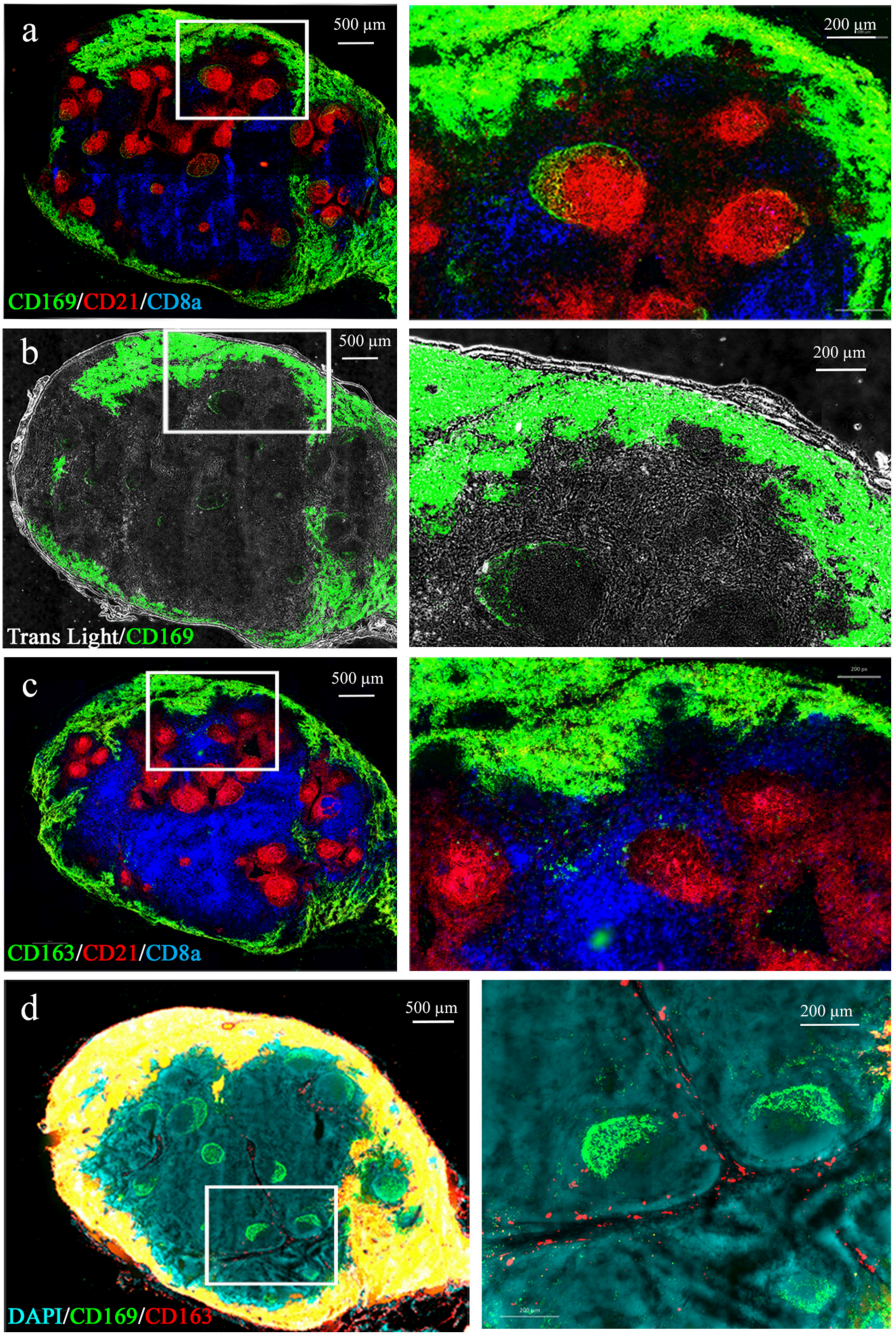
Macrophages localization in the porcine tracheobronchial lymph nodes. Whole lymph node cryosections were immunostained for B **(a)** and T **(b)** cell zones using anti-CD21 (red) and anti-CD8α (blue) antibodies, respectively. A third antibody directed against CD169 **(a,b)** or CD163 **(c)**, was added. In **(b)** the transmitted light was also recorded. **(d)** CD163/CD169 costaining. Images are representative of at least 3 LN from different animals. Whole LN pictures are individual images from 10x or 20x objective acquisitions, assembled using axiovision® software (Zeiss).

We first identified swine LN MΦ according to their LN localizations with regard to B and T cell areas. The anti-CD21b antibody B-Ly4 mainly stains immature pre-class-switched B cells (34) and was used to localize B cell follicles. Anti-CD8α expressed on CD8 T cells and memory/activated CD4 T cells in swine (35) was used to identify the T cell areas (Figures 1a,c). As previously observed in swine (26), T cell areas are diffused, whereas B cell follicles are precisely delineated. CD169 and CD163 markers previously used to identify swine LN MΦ (27) and mouse LN MΦ were used [for review (8)]. As expected LN from conventionally reared animals (Houdan slaughterhouse) frequently presented several well-developed B cell follicles, whereas control-reared animals from INRA heard presented fewer and smaller follicles (data not shown). We observed CD169 expression at the periphery of the LN (Figure 1a) in an extended subcapsular location (Figure 1b). These CD169 subcapsular staining colocalized with CD163 staining (Figures 1a,c,d). Because of the reverse structure of the pig LN, these peripheral cells are localized next to the efferent vessels. Interestingly, CD169 was also expressed in close association with the B cell follicles (Figure 1a). CD169^pos^/CD163^neg^/CD21^neg^ cells were situated on a thin band at the periphery of the CD21-positive follicles (Figure 1a, green perifollicular crescent) whereas CD169^pos^/CD163^neg^/CD21^pos^ cells were identified in a more diffuse “crescent shape” intrafollicular area (Figure 1a, yellow intra-follicular staining). Finally, CD169^neg^/CD163^pos^ cells were present in the medulla, tightly associated with the lymphatic cords, both in the LN parenchyma as well as in the sinus (Figure 1d, red staining).

### Three Different MΦ Cell Types Can Be Identified in the Tracheobronchial LN

**Figure 2:**
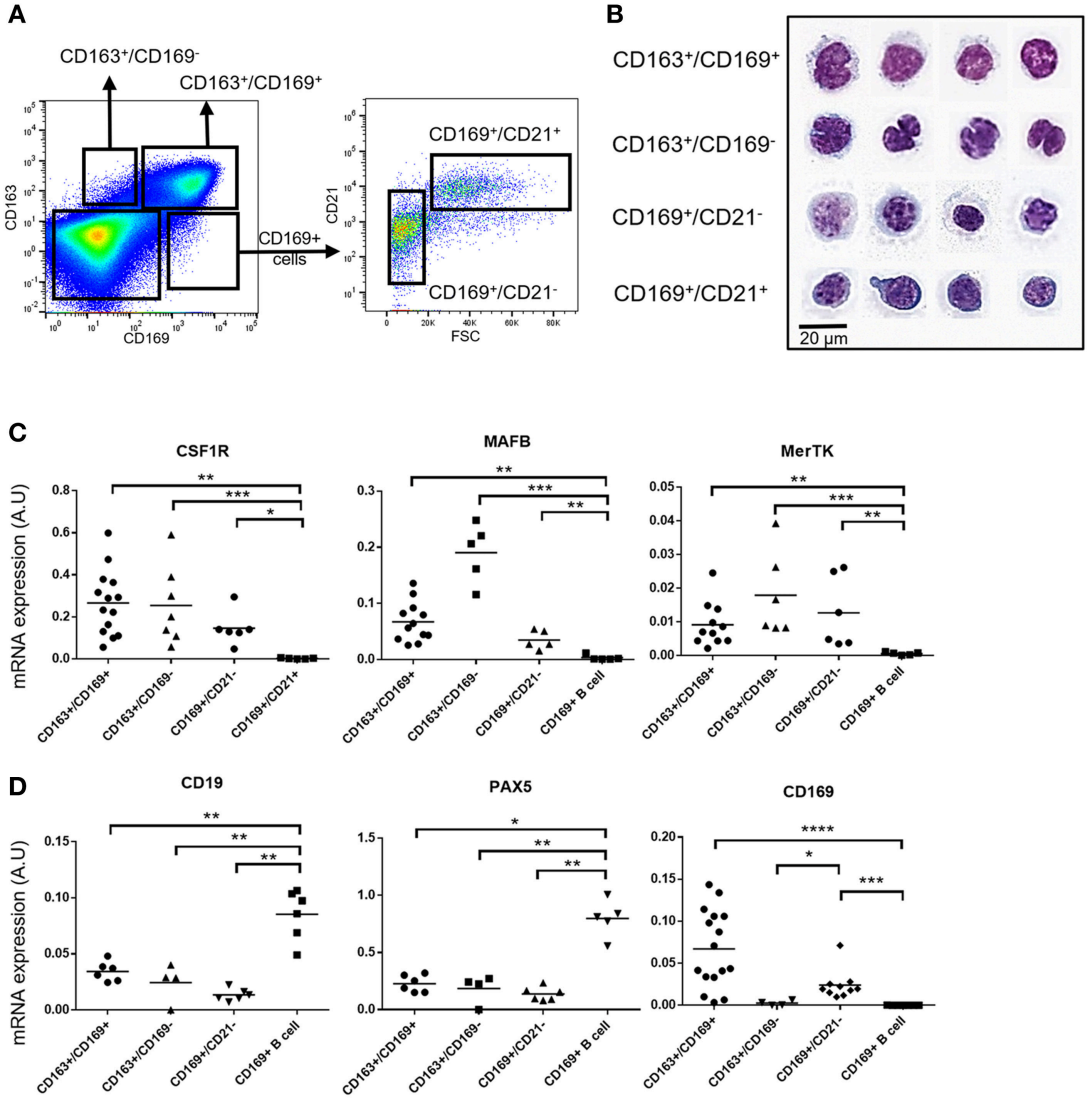
Tracheobronchial lymph nodes macrophages identification. **(A)** Representative dot plots of the LN macrophages populations by FACS analysis using CD163, CD169, and CD21. The first gates on cells, singlets and live cells are the same as the ones depicted in Supplementary Figure 2a. Four populations were defined: CD169^+^/CD163^+^ efferent macrophages (effMΦ), CD169^−^/CD163^+^ cord macrophages (cordMΦ), CD169^+^/CD163^−^/CD21^−^ perifollicular macrophages (PFMΦ) and CD169^+^/CD163^−^/CD21^+^ CD169^pos^ B cells. **(B)** Sorted cells were stained by a May-Grünwald-Giemsa (MGG) coloration for morphological analysis. Data are representative of 3 independent experiments. **(C,D)**, mRNA expression of macrophagic **(C)** and B cell **(D)** specific genes in the sorted populations by RT-qPCR. Arbitrary unit: A.U. represents 2^−Δ*Ct*^, using RPS24 as reference gene. The Mann-Whitney's test was performed. *P-values* * < 0.05, ** < 0.01, *** < 0.001, **** < 0.0001.

A flow cytometry analysis of enzyme-digested LN was performed to isolate the 4 populations identified using microscopy. The 4 populations were distinguished using a CD163/CD169/CD21 and FSC gating and sorted as described in Figure 2A, followed by MGG staining (Figure 2B) or by RT-qPCR (Figure 2C). CD163^pos^/CD169^pos^ cells presented a rounded or slightly indented nucleus with coarse chromatin and abundant clear vacuolated cytoplasm, in agreement with a macrophagic phenotype (Figure 2B). Moreover, RT-qPCR analyses revealed that these cells expressed the macrophagic CSF1R, MAFB, and MerTK genes (Figure 2C). Because of the reverse structure of the pig LN, these cells located at the periphery are next to the efferent vessels. Thus, we called them efferent MΦ (effMΦ). CD163^pos^/CD169^neg^ cells displayed abundant clear vacuolated cytoplasm and a large deeply indented nucleus with lacy chromatin similar to blood monocytes and to the MCM from murine LN (11). They expressed similar levels of CSF1R and MerTK than effMΦ but higher levels of MAFB (Figure 2C). According to their association with the cord vessels, these cells were called cord MΦ (cordMΦ).

CD163^neg^/CD169^pos^/CD21^neg^ cells displayed a blue-gray cytoplasm with a grainy texture surrounding a large vesicular or slightly indented nucleus with coarsely clumped chromatin (Figure 2B). These cells expressed CSF1R, MAFB, and MerTK genes (Figure 2C). According to their morphology and their localization at the periphery of the follicle, these cells were called perifollicular MΦ (PFMΦ). CD163^neg^/CD169^pos^/CD21^pos^ cells displayed a high nucleo-cytoplasmic ratio with a large round nucleus with coarse and dense chromatin and a nucleolus surrounded by a perinuclear halo and a rim of basophilic cytoplasm, looking very similar to porcine bone marrow preB cells (36), in strong support with an immature B cell identity. Moreover, their localization in the periphery of the follicle (Figures 1a,d), reminds the dark zone localization of centroblasts in mouse and human [for review see (37)]. In agreement with their immature B cells microscopic profile, these cells expressed CD19, a component of the B cell receptor complex, and PAX5, a regulatory gene involved in the somatic hypermutation process (38) (Figure 2D) but none of the macrophagic markers tested (Figure 2C). The cells were subsequently named CD169^pos^ B cells. Although CD169 protein expression was detected by FACS (Figure 2A) and microscopy (Figures 1a,d), CD169^pos^ B cells did not express detectable levels of CD169 mRNA (Figure 2D). Conversely, CD169 mRNA quantification in effMΦ (CD169^pos^), cordMΦ (CD169^neg^) and PFMΦ (CD169^pos^), was in agreement with cytometry and microscopy data (Figure 2D).

### PFMΦ Cells Cap the B Cell Follicles and Interact With Intrafollicular CD169^pos^ B Cells

**Figure 3:**
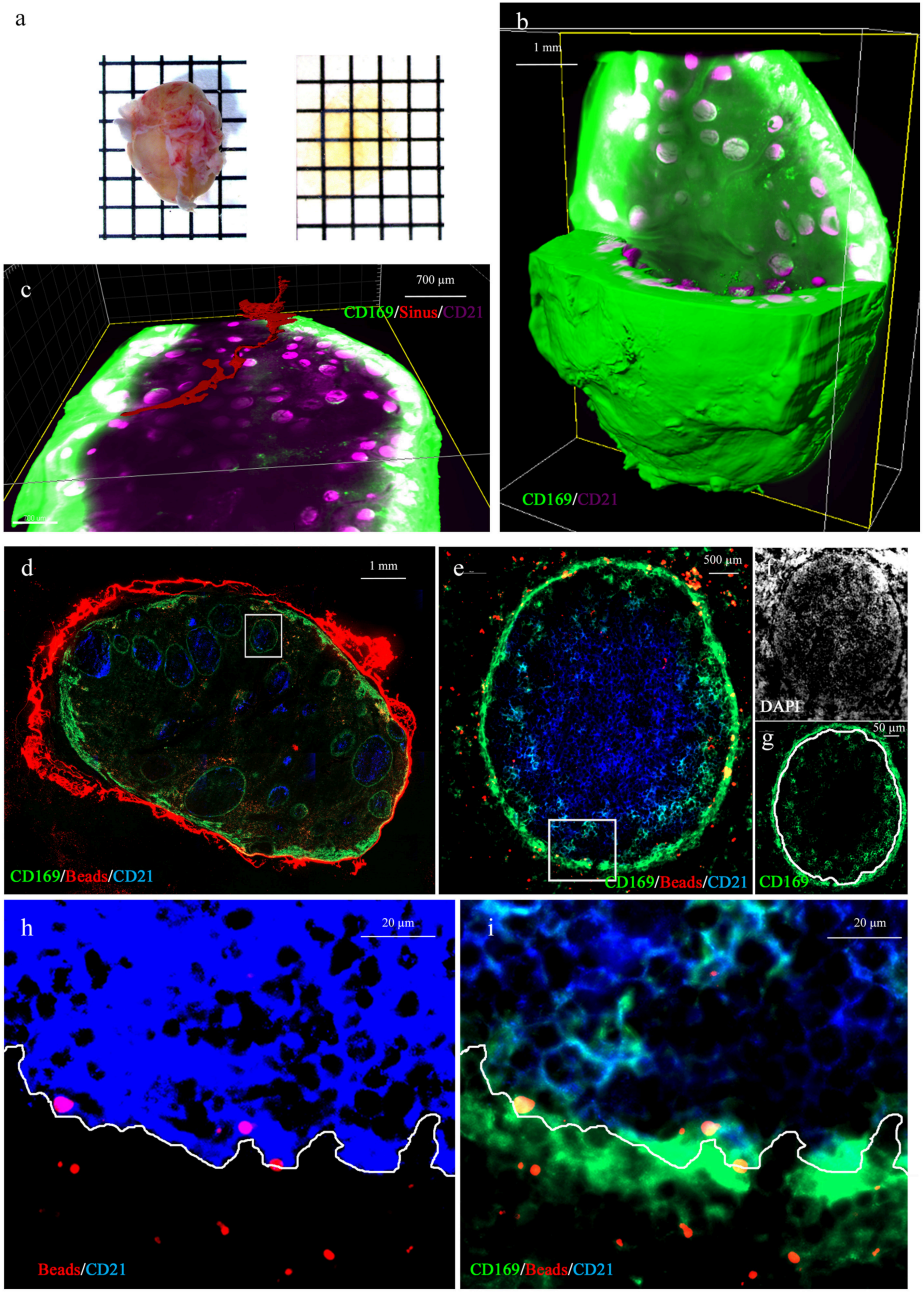
Whole 3D LN imaging and antigen fate. **(a)** Whole lymph node before (left) and after (right) clearing. **(b,c)** 3D reconstruction of a whole transparised LN (green, CD169, magenta CD21 staining). The green channel has been saturated in peripheral effMΦ to better discriminate CD169^low^ PFMΦ and to reveal the tissue autofluorescence, in order to visualize the sinus lumen. **(c)** Tracking of the lumen of one continuous afferent lymphatic/parenchymal sinus (in red). **(b,c)** are static depictures of Supplementary Movies 1, 2, imaged using a light-sheet microscope. **(d–i)** Tracheobronchial explant was injected with 0,1 μm red fluorescent beads and incubated for 30 min at 37°C before preparation for staining with anti-CD169 (green) and anti-CD21 (blue) and DAPI (white). **(d)** Whole draining LN (10x objective). **(e)** One selected follicle from the draining LN [White square in **(d)**] were analyzed. Yellow spots represent red beads co-localized with CD169-positive cells. Light blue cells are CD169^pos^/CD21^pos^ follicular B cells. The white square delineates the area depicted in **(h,i)**. **(f,g)**, single color (white: DAPI, green: CD169) depicture of image **(e)**. The DAPI **(f)** picture allowed us to delineate the follicle limit [white line in **(g)**]. **(h)** DAPI and saturated CD21 staining allowing the delineation between intrafollicular and extrafollicular areas (white line). **(i)** Same picture as in **(h)**, using different image treatment in order to observed co-localisations of CD169, CD21 and Beads. Some red beads situated inside the follicle are depicted in yellow because of their collocalisation with PFMΦ (green) and/or CD169^pos^ B cells (blue and green, depicted in light blue). Pictures **(e–i)** were obtained using a 63x objective.

To investigate the relationship between the PFMΦ and the B cell follicles we proceeded to whole LN transparisation (Figure 3a), combined with CD169/CD21 immunostaining and imaging using fluorescent light sheet microscope. This allowed to reconstitute the whole LN 3D structure (Figure 3b and Supplementary Movie 1). This tracheobronchial LN, sampled from a conventionally reared animal, contained 539 B cell follicles (Supplementary Movie 2), each of them being individually associated with a PFMΦ area (Supplementary Movie 1). The afferent central entry appeared composed of a collection of smaller afferent vessels which separated from each other while entering deeper in the LN parenchyma (Supplementary Movie 1). One of this afferent vessel and its continuous sinus was tracked (in red, Figure 3c and Supplementary Movie 2). The sinus appeared interconnected with other sinuses along its parenchymal trail to finally pour in the efferent subcapsular sinus.

The 3D rendering of follicles acquired at higher resolution delineated a PFMΦ area forming a semi-spherical structure that cap the B cell follicle on one of its sides (Supplementary Movie 3).

To identify the MΦ taking in charge particulate antigens drained through afferent lymphatics, we injected *ex vivo* red fluorescent 0.1 μm beads in the tissue surrounding a tracheobronchial LN. Injected beads were allowed to drain for 30 min at 37°C. The draining LN was then sampled and immunostained for CD169 and CD21 expressions. Beads were present in effMΦ and PFMΦ but not in cordMΦ (Figure 3d). Zooming on B cell follicles, beads were mostly associated with PFMΦ although some signal was observed deeper in the follicle associated with CD169^pos^ B cells (Figure 3e). By referring in DAPI staining, PFMΦ appeared clearly situated in the space between the follicle and the LN parenchyma, reminiscent of mouse LN subcapsular sinus space (Figures 3f,g). To precisely set the limit between the CD21^pos^ cells and the PFMΦ, a view of the interface between intrafollicular B cells and PFMΦ is depicted in Figure 3h with saturated CD21 staining. Once this limit affixed on the CD169/CD21 co-staining (Figure 3i), several beads appeared situated at the contact between PFMΦ protrusions and intrafollicular CD169^pos^ B cells (Figure 3i). Thus, PFMΦ and CD169^pos^ B cells closely interact with antigen drained from the peripheral tissue, at the frontier of the B cell follicle.

### Identification of Five LN B Cell Differentiation/Activation Stages

**Figure 4:**
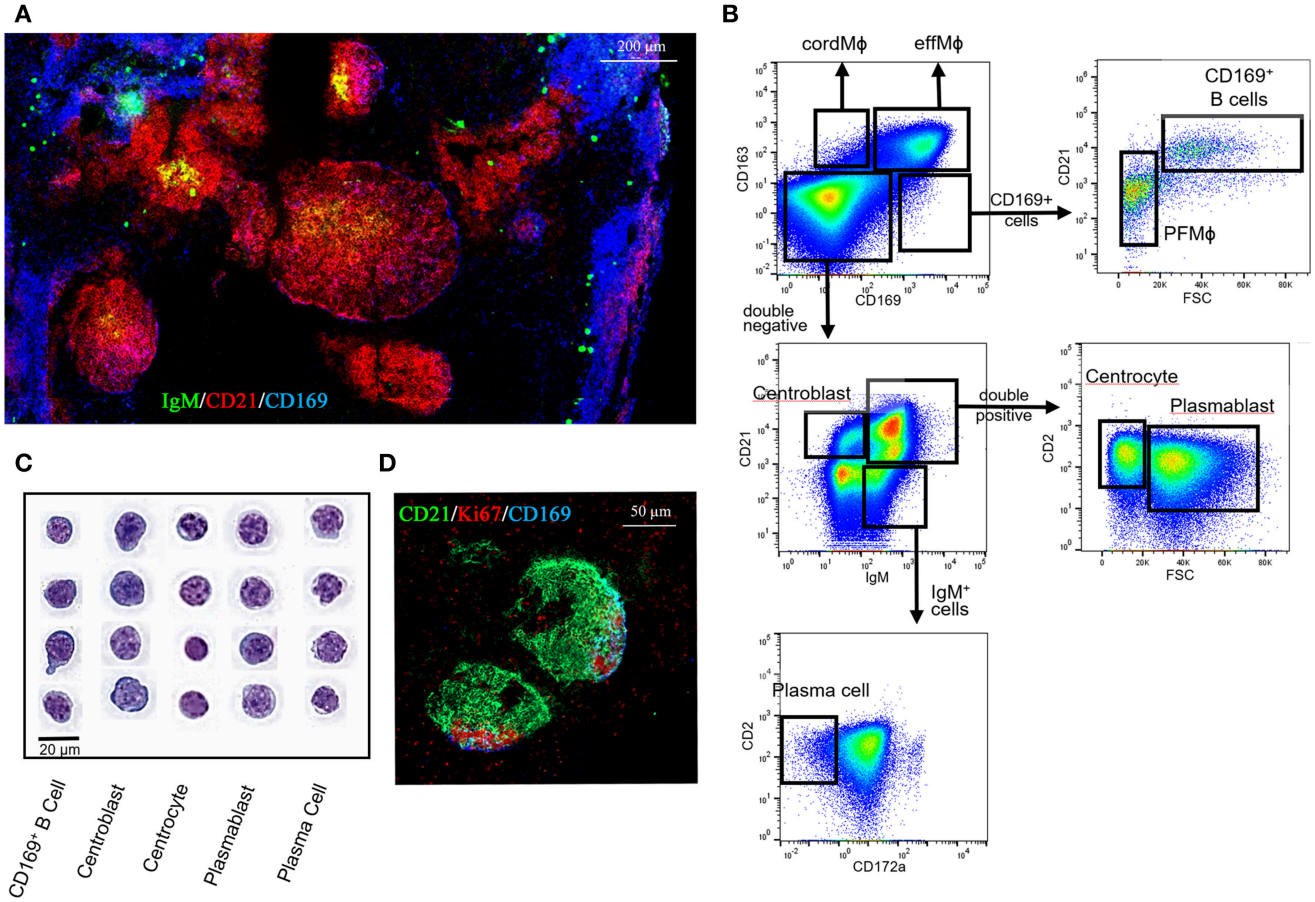
B cell populations characterization in the tracheobronchial lymph nodes. **(A)** Lymph node cryosections were immunostained for IgM (green), CD21 (red) and CD169 (blue). **(B)** Representative dot plots of the LN B cell populations by FACS analysis using CD163, CD169, CD21, CD2, and IgM markers. **(C)** Sorted cells were spun onto slides and stained by MGG coloration for morphological analysis. **(D)** Lymph nodes cryosections were immunostained for CD21 (green), Ki67 (red), CD169 (blue). Data are representative of 3 independent experiments.

The CD21, IgM, and CD2 markers, previously proposed by Sinkora et al. (39) for porcine bone marrow B cell development analysis, were used to better integrate CD169^pos^ B cells across the LN B cell differentiation steps. The CD169^pos^/CD21^pos^ follicular B cells (Figure 4A, purple cells) did not express IgM and were mixed with CD21^pos^/CD169^neg^/IgM^neg^ B cells (red cells) in the intrafollicular area contiguous with PFMΦ (blue cells). IgM^pos^/CD21^pos^ cells (yellow cells) were localized in the center of the follicle. Finally, IgM^pos^/CD21^neg^ cells (green cells) were rarely present in the center of the follicles, but well represented in extrafollicular area, as well as in the LN periphery, in direct contact with effMΦ (Figure 4A). Thus, this first overview of LN B cells phenotypic localization is in agreement with CD21^pos^/IgM^neg^ cells (CD169 positive and negative) being dark zone-localized centroblasts, IgM^pos^/CD21^pos^ cells being light zone centrocytes and plasmablasts and IgM^pos^/CD21^neg^ cells being mostly extrafollicular mature plasma cells.

We completed this microscopic study by FACS analysis using the panel utilized for MΦ identifications, which was complemented with the myeloid marker Sirpa/CD172a and the B cell markers CD21, IgM and CD2 (Figure 4B). The different cell-types were sorted as described in Figure 4B and stained using MGG coloration (Figure 4C). Like CD169^pos^ B cells, centroblasts displayed a large, round nucleus with multiple peripheral nucleoli surrounded by a rim of basophilic cytoplasm. Centrocytes displayed a small, round nucleus with clumped chromatin and with scant cytoplasm. Plasmablasts displayed some heterogeneity; their size was intermediate to large with scant to moderate slightly basophilic cytoplasm and a vesicular nucleus with reticular chromatin. Plasma cells demonstrated an intermediate-sized nucleus with reticular chromatin and a rim or just a crescent of clear cytoplasm with a prominent juxtanuclear archoplasm.

Using Ki67 staining we identified proliferating B cells that appeared mostly localized in the follicle area contiguous to CD169^pos^ PFMΦ, in agreement with the phenotype and dark zone-localization of CD169^pos^ B cells and centroblasts (Figure 4D).

**Figure 5:**
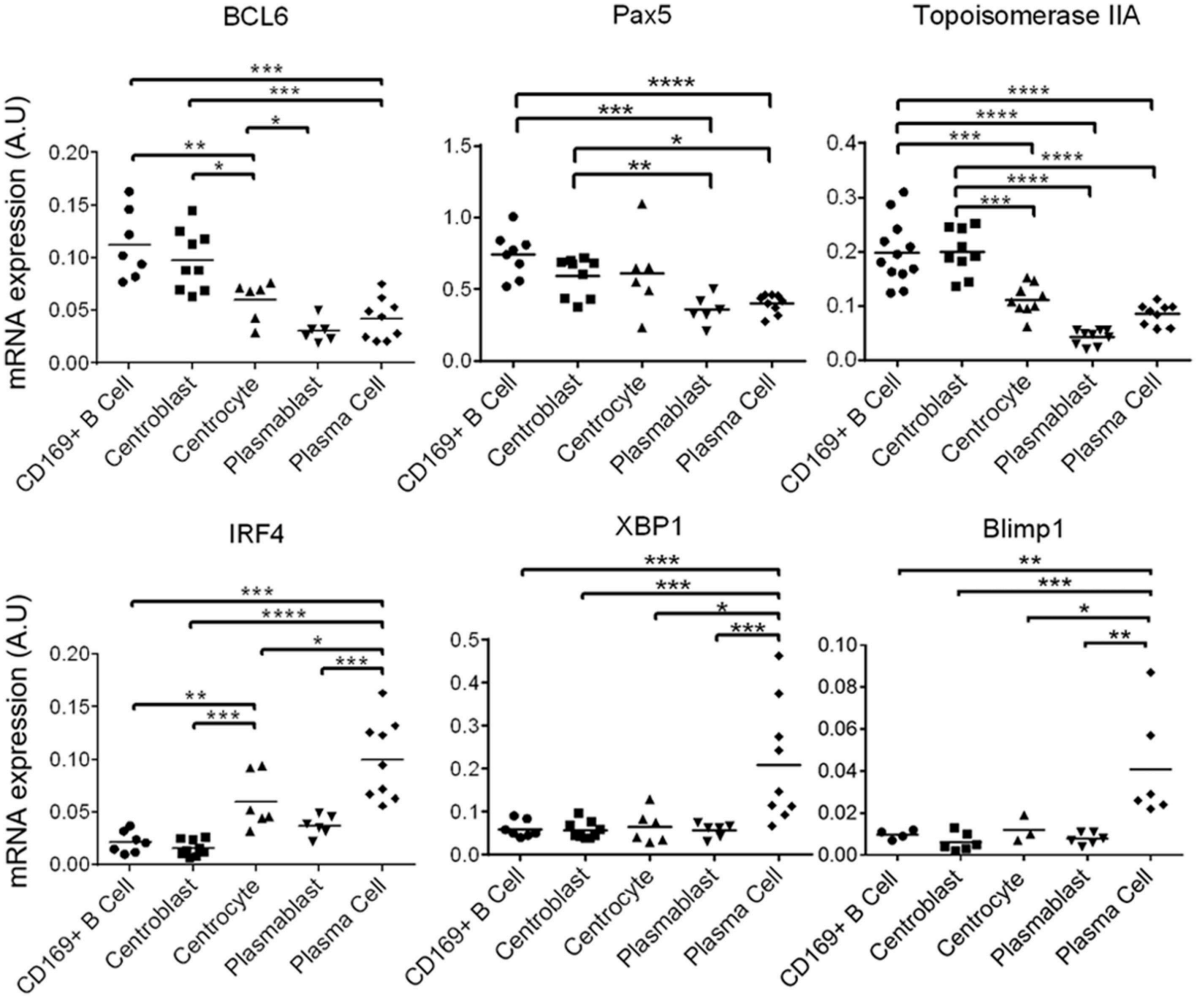
Identified tracheobronchial lymph nodes B cell populations recapitulate the B cell differentiations steps. mRNA expression by RT-qPCR of B cell differentiation genes, in the sorted populations defined in Figure 4B. Arbitrary unit: A.U. represents 2^−Δ*Ct*^, using RPS24 as reference gene. The Mann-Whitney's test was performed. *P-values* * < 0.05, ** < 0.01, *** < 0.001, **** < 0.0001.

The sorted B cells were characterized by analyzing B cell-transcription factors genes expression by RT-qPCR (40) [for review see (37)]. CD169^pos^ B cells and centroblasts expressed significantly more BCL-6 (Figure 5) than all the other B cells. BCL-6 is a master transcription factor expressed specifically in centroblasts (40) and involved in the inhibition of their differentiation into plasma and memory B cells (40, 41). Centrocytes expressed median levels of BCL-6, PAX5, and IRF4 as expected for these cells at an intermediate differentiation stage (Figure 5). As expected, plasmablasts and plasma cells expressed the lowest levels of BCL-6 and PAX5, whereas plasma cells expressed the highest levels of IRF4 (42), XBP1 (43, 44), and Blimp1 (45, 46) which are known markers of terminally differentiated B cells (Figure 5). CD169^pos^ B cells and centroblasts (CD21^pos^/IgM^neg^) expressed the proliferation markers Topoisomerase IIA (Figure 5) and Cyclin B2 (Supplementary Figure 1b) at significantly higher levels than all the other B cells, which is consistent with the expression of Ki67 in the follicular area contiguous with PFMΦ.

In conclusion, the phenotype, the localization, the expression of commonly used transcription factors and the proliferation status consistently defined five B cell maturation stages. To note, we confirmed here that the CD169^pos^ B cells were strongly related to centroblasts, and might be an early step of this differentiation stage.

### *In vivo* PRRSV Interact With EffMΦ and PFMΦ

*In vivo* infections were performed to explore the mechanisms used by PRRSV to persist in the porcine LN, and its impact on the follicular B cell maturation process.

In a first experiment, 4 animals were infected with Lena PRRSV and tracheobronchial LN were collected 10 dpi. At the time of this preliminary experiment we did not yet distinguish PFMΦ from CD169^pos^ B cells, we thus sorted one single CD169^pos^/CD163^neg^ population. We also sorted LN cDC2 as a myeloid negative control of infection (23) as described in Supplementary Figure 2a. The sorting strategy was validated by RT-qPCR of key specific genes (Supplementary Figure 2b). As previously validated (23), we measured cell-associated PRRSV by RT-qPCR on viral RNA normalized using RPS24 reference gene (2^−Δ*Ct*^). PRRSV RNA was detected in effMΦ (10 dpi) (Supplementary Figure 2c) in agreement with effMΦ population decrease proportion among total LN live cells (Supplementary Figure 2d), whereas a weaker expression was detected in the pooled PFMΦ/CD169^pos^ B cells (Supplementary Figure 2c), with no significant decrease of this last composite population (Supplementary Figure 2d).

**Figure 6:**
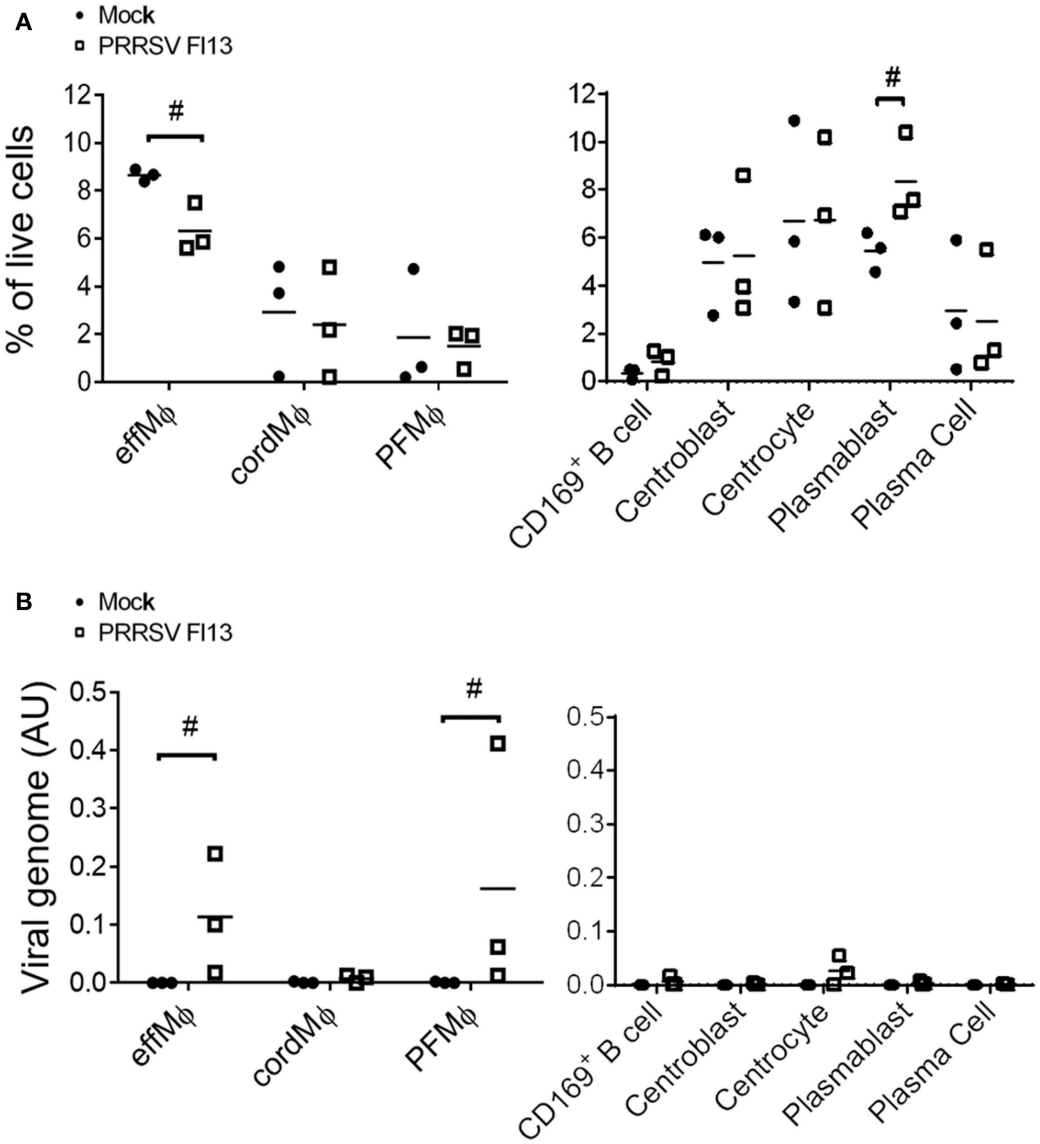
effMΦ and PFMΦ interact with FL13 PRRSV. *In vivo* infected and control tracheobronchial LN cells were sorted using previous gating strategy defined in Figures 2A, 4B. **(A)** Evolution of population upon infection were analyzed as a percentage of each population among the total LN live cells extracted from flow cytometry data. **(B)** Detection of viral RNA were performed on sorted cells by RT-qPCR. ^#^highlights when the infected animals were all upper or lower than the controls.

Realizing that the CD163^neg^/CD169^pos^ population was composed of mixed B and macrophagic cells we then performed a second *in vivo* infection in which we discriminated PFMΦ from CD169^pos^ B cells. Moreover, to ascertain that our first results could be extended to different strains and times post-infection, we then used Flanders 13 strain and sacrificed the animals 5 dpi. Three infected animals were sacrificed, tracheobronchial LN cells were isolated and analyzed using the same gating as in Figure 4B. Upon infection, a proportional decrease of effMΦ and an increase of plasmablasts were observed while no change in the proportions of the other cell types occurred (Figure 6A). The populations were sorted and viral RNA content was measured by RT-qPCR. In agreement with the preliminary experiment, PRRSV RNA was detected in effMΦ (Figure 6B) [Ct = 29.0 ± 1.3 (SD)] but not in the cordMΦ (Ct = 35.6 ± 0.7). Interestingly PFMΦ (Ct = 27.8 ± 1.5) but not CD169^pos^ B cells (Ct = 35.8 ± 2.6), were positive. No PRRSV RNA was consistently detected in the B cell compartment. Thus, 10 days post-Lena infection and 5 days post-FL13 infection we observed cell depletion and viral RNA in effMΦ as well as viral RNA in PFMΦ.

### *In vivo* PRRSV Infection Triggers Upregulation of BAFF in EffMΦ and of BCl6 in CD169^pos^ B Cells

**Figure 7:**
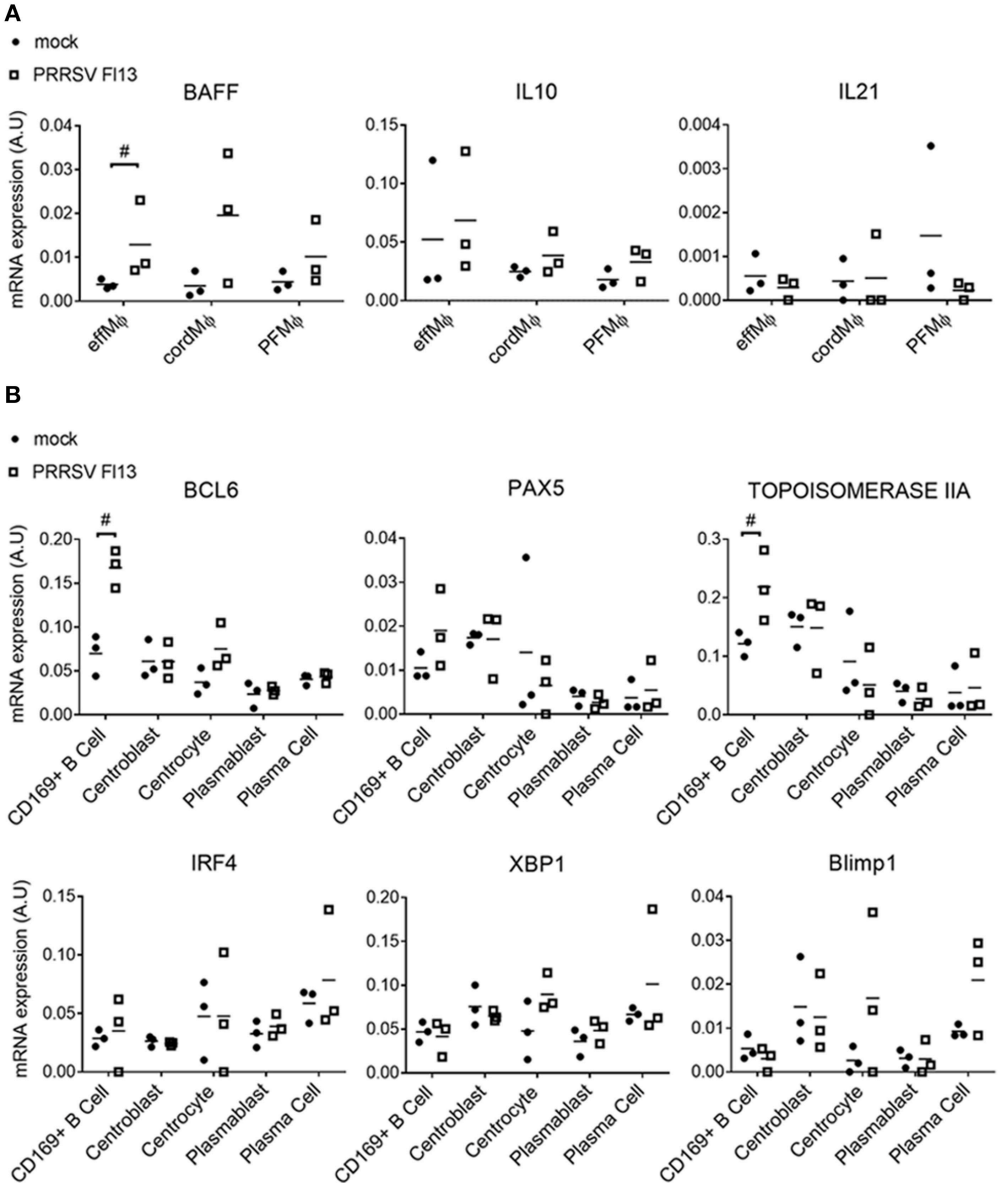
PRRSV/FL13 infection triggers BAFF upregulation in macrophages and BCL-6 upregulation in CD169^pos^ B cells. Macrophages and B cells were sorted from *in vivo* infected tracheobronchial LN at 5 days post FL13 PRRSV infection. **(A)** mRNA expression of cytokines was measured by RT-qPCR. **(B)** mRNA expression of B cell transcription factors involved in the different B cell differentiation steps was measured by RT-qPCR. ^#^highlights when the infected animals were all upper or lower than the controls.

The expression of cytokines involved in B cell survival/maturation, IL-10, IL-21 and BAFF were measured in LN MΦ upon FL13 infection. PRRSV infection induced the transcriptomic upregulation of BAFF in effMΦ (Figure 7A) but did not modify IL-10 and IL-21 transcriptional expressions.

We then tested if PRRSV infection impacted the B cell differentiation program by measuring the expression of 5 B cell transcription factors (BCl6, Pax5, IRF4, XBP1, Blimp1). Strikingly, CD169^pos^ B cells but not CD169^neg^ centroblasts or other B cell differentiation stages, presented a 2.4-time increase in BCL-6 expression, paralleled with a 1.8-fold increase in Topoisomerase IIA (Figure 7B).

## Discussion

In mammalians, the lymph node is the control center of the adaptive immune response, that's why efforts have been developed recently in the mouse model to better describe its structure/function relationships, leading to a better understanding of LN cells complex relationships, in steady states and infectious situations (47–50). Although several swine pathogens, among them PRRSV, are known both to persist in the LN and to strongly delay the onset of an effective immune response, the structure of the porcine lymph node and its striking inverted peculiarity had never been precisely investigated.

Herein, we first explored the inverted structure of the pig LN according to LN MΦ populations and B cell maturation stages. The effMΦ population had a subcapsular localization at the exit of the LN, whereas human and mice SCS MΦ are positioned at the entrance of the LN. Because of this distinction, pig subcapsular MΦ are expected to be endowed with different functions compared to mouse and human SCS MΦ. Due to their localization before the efferent lymphatic, we considered them as the likely functional equivalent of the mouse MSM, localized in the medullary sinus, just before the efferent lymphatic vessel. Indeed their phenotype (CD169^pos^/CD163^pos^/CD21^pos^) corresponds to the CD169^pos^/CD163^pos^ phenotype known for MSM (8) rather than to the phenotype of murine SCS (CD169^pos^/CD163^neg^) (51) (Table 2). In mouse, these MSM have been proposed to play a role in the clearance of the lymphatic fluid before its exit to the main blood circulation (8). CordMΦ are clearly positioned along the medullary cords with a phenotype similar to the mouse MCM (CD163^pos^/ CD169^neg^). Finally, PFMΦ appeared to be the equivalent of murine SCS MΦ (Table 2). They presented a CD169^pos^/CD163^neg^/CD21^neg^ phenotype compatible with mouse SCS MΦ and with the porcine CD169^pos^ spleen MΦ (27), which are the likely counterpart of metallophilic marginal zone MΦ, i.e., the spleen equivalent of SCS (52). We brought here evidences that PFMΦ are positioned at the interface between afferent lymph and B cell follicles and that they are endowed with the capacity to bind and transfer antigens from the sinus to the intrafollicular compartment as described for murine SCS MΦ (10, 53–55). We did not find evidence neither of tangible body MΦ, nor of T cell zone MΦ, probably because of their absence of expression of CD169 and CD163, the two markers we used here for porcine LN MΦ identification.

**Table 2:** Tracheobronchial lymph nodes macrophages comparison between mouse and swine.

		**Mouse**	**Swine**
Antigen entry (afferent)	Name	SCS MΦ	PFMΦ
	Location	Subcapsular & perifollicular	Perifollicular
	Phenotype	CD169^+^/CD163^−^	CD169^+^/CD163^−^
	Main function	Antigen transfer into B follicle	/
Lymphocytes exit (efferent/HEV)	Name	MCM	cordMΦ
	Location	Medullary cord	Cord
	Phenotype	CD169^−^/CD163^+^	CD169^−^/CD163^+^
	Main function	Plasma cells maturation	/
Fluid exit (efferent)	Name	MSM	effMΦ
	Location	Medullary sinus	Subcapsular
	Phenotype	CD169^+^	CD169^+^/CD163^+^
	Main function	Lymph clearance before exit	/

We observed a CD169^pos^ B cell population positioned inside the B cell follicles at the contact with PFMΦ. Except their exhibition of CD169, these cells are CD21^pos^/IgM^neg^/CD2^neg^. They are proliferating and their MGG phenotype as well as transcription factors expression (BCL-6 and PAX5) are characteristic of the centroblastic stage. Interestingly these cells exhibit CD169 proteins at their surface without detectable mRNA. This discrepancy between mRNA and protein expression associated with their observed close interaction with PFMΦ supports the acquisition of CD169 molecules by intrafollicular CD21^pos^ centroblasts through trogocytosis upon intimate contact with CD169-expressing PFMΦ. This membrane material exchange gives credit to the possibility of antigen transfer from PFMΦ to CD169^pos^ B cells. Indeed, the B cell interaction with antigen-bearing PFMΦ in the porcine lymph node appears similar to the previously described murine SCS MΦ/B cells interaction in their relay on antigen transport from the capsule to the follicle (10, 53, 54). Interestingly, in pig, these antigen-transporting B cells belong to the centroblast stage, whereas in mouse, naïve B cells are supposed to fulfill this task (55). Centroblasts are the cells defining the dark zone, this means that, differently from mouse SCS MΦ which are in contact with the centrocyte-occupied light zone, porcine PFMΦ are in contact with the centroblast-occupied dark zone. It remains to be explored how this striking difference may affect the follicular functions of the porcine LN. After profiling the steady state of LN cells, we analyzed the different MΦ and B cell populations upon PRRSV infection. According to their PRRSV RNA level associated with their depletion upon infection, effMΦ are likely productively infected by both FL13 and Lena viruses. Because of effMΦ localization, and since their depletion might preclude them to play their role of efferent lymph scavenger, this infection might increase the dumping of viral particles from the LN to the blood circulation, thus contributing to the viremia. Compared to effMΦ, PCMΦ and cDC2 did not present consistent PRRSV RNA levels. Interestingly, PFMΦ presented comparable viral RNA load as effMΦ, whereas PFMΦ did not express the major PRRSV entry receptor CD163. For several viruses (53), it has been described that SCS (the *bona fide* PFMΦ murine counterpart) are endowed with the capacity to capture lymph-borne viruses for presentation to follicular B cells, being infected or not. Unfortunately, the scarcity and the fragility of PFMΦ did not allow us to unambiguously test their capacity to replicate PRRSV *in vitro*. However, the absence of CD163 expression associated to the fact that PFMΦ did not experience depletion upon *in vivo* infection are in agreement with the occurrence of a viral interaction without productive infection.

Concerning the modulation by PRRSV of cytokine expressions related to B cell homeostasis, BAFF was found to be upregulated in LN MΦ upon infection whereas levels of IL21 and IL10 were unchanged. Although limited, this information is in agreement with an adequate survival environment for activated B cells. In the B cell compartment, only the CD169^pos^ B cells, in close contact with PFMΦ, presented increase of BCL-6 expression and proliferation. Although it is difficult to interpret these data without a comparison with another respiratory virus triggering a normal neutralizing antibody response such as *influenza*, the strict limitation of BCL-6 upregulation and proliferation in CD169^pos^ B cells led us to hypothesize a blocking of early centroblasts at this immature low affinity state. Interestingly, in germ free piglets PRRSV infection triggers a non-antigen selective expansion of B cells in all immunoglobulin classes, suggestive of a PRRSV-induced defect in the B cells maturation process (56). This blocking might contribute to the PRRSV-induced defect in the B cell maturation process observed by Sinkora et al. (56), linked with the production of hydrophobic binding sites autoimmune-like antibodies (57). These observations might be related to similar B cell maturation defects (58) as well as low-affinity autoimmune antibodies appearance (59) observed in HIV infections, another single strand positive RNA virus, in which the gp120 virus envelope protein act directly on B cell membrane proteins to trigger a polyclonal, non-protective B cell response (60). In conclusion, we observed in porcine tracheobronchial LN a strong PRRSV signal associated with depletion of the effMΦ, in agreement with a productive infection that can participate to the viremia. Conversely, interaction of PRRSV with PFMΦ might favor a direct or indirect action of the virus on CD169^pos^ B cells, leading to their blocking in a BCL6^high^ state.

The fine description of the inverted porcine LN MΦ and B cell differentiation steps will open the possibility to visualize the action of PRRSV and other porcine viruses, such as the African swine fever virus, the classical swine fever virus and the porcine circovirus type 2 in this main place of the adaptive immune response initiation. Finally, an in depth understanding of inverted porcine LN structure may also allow to look from a new point of view on the “normal” structure of human and mice lymph nodes and may help veterinarians to better understand the immunology of endangered species such as rhinoceros and dolphin.

## Ethics Statement

The animal experiments were authorized by the French Ministry for Research (authorization no.2015051418327338 and no.2015060113297443 respectively) and approved by the national ethics committee (authorizations no.09/07/13-1 and no.07/07/15-3).

## Author Contributions

ElB, EC, EdB, and NB processed the samples and performed the *in vitro* and *ex vivo* experiments. MF and CL performed the transparisation, the immunological staining, and the image analysis of the whole LN. SR, ElB, and NB performed, acquired and analyzed the regular LN immunostaining. JP, AP, PR, OBour, and OBoul performed the *in vivo* experiments. ElB, NB, and MB performed the cell-sorting. TL provided strong support for MGG and immunofluorescence images interpretation. OBour and OBoul supervised the *in vivo* experiments. ElB, EC, EG, and IS-C provided thorough discussions and critical manuscript reading. EG, IS-C, and NB provided financial supports. NB designed experiments and wrote the manuscript.

### Conflict of Interest Statement

The authors declare that the research was conducted in the absence of any commercial or financial relationships that could be construed as a potential conflict of interest.
