# Supplementary material for: Macrophage-B Cell Interactions in the Inverted Porcine Lymph Node and Their Response to Porcine Reproductive and Respiratory Syndrome Virus
Source: Front Immunol. 2019 May 3;10:953. doi: 10.3389/fimmu.2019.00953 (PMC6510060; doi:10.3389/fimmu.2019.00953)
Supplement: Supplementary file 4 [file Data_Sheet_1.PDF]

Gene	Sequences (5'-3')	Gene ID	Size pb	Efficiency
CSF1R	F° CGGTATGTGCCCGTGTTTTG<br>R° CGATGCGTGAGCAATGTCAG	100517086	86	0.96
MAFB	F° TGCGTTCTTTAGACCAATATGTTATGT<br>R° CACCAATAACTCGCCCGCTAT	100518227	71	0.99
MerTK	F° CCGAACTCTGTAATCGCTTCTTG<br>R° TGCACTTCCGCCGTGACTA	100519652	65	0.74
CD169 /SIGLEC1	F° CAGGGGGAGTATGTGTGCTC<br>R° CCTGGGTAGATTGGGGTTCAC	397623	441	0.98
Topoisomerase IIA / TOP2A	F° TCAGCCTGGCCTTTAGCAAA<br>R° TCGTTGCCGTCTATCTTCCAT	396917	67	0.92
Cyclin B2 / CCNB2	F°AGTTTAGGCTGCTGCAGGAAAC<br>R°TGGCTGAACCTGTAAAAATCGA	100135668	70	0.86
Ki67 / MKI67	F° GCAGACGCTACCGCTCATC<br>R° GCCAAGTAACTAAGGACAGCTCTGA	102164100	72	0.74
CD19	F° CAAGATGCTGACACCTATCACTGTAA<br>R° TGACCGAGCAGTGACCTTCA	397669	74	1.00
BCL-6	F° CGAGAAGTGTAACCTGCATTTCC<br>R° CGTGCTTCTGGCGCAAGT	100156549	64	0.91
PAX5	F° TCACAGCATAGTGTCCACGG<br>R° CCGGCAGTGAGTGACCAT	100520167	182	0.98
IRF4	F° ACTCCCAGCTCAGGTTCACAA<br>R° GGACGAACTCCCTCCATCCT	100144625	65	1.00
XBP1	F° AGCACAGAAGCCTGTGGTTC<br>R° ACAACCTGCATGTCCTAGACC	100217386	144	0.95
Blimp1 /Prdm1	F° TGAAACTCCACAAGCGCCTAC<br>R° CCTTCAGATGGACCTTGAGGC	12142	101	0.96
PU-1 /SPI1	F° TCCCCCCTCAGCCATCA<br>R° GCGTTTGGCGTTGGTAGAGA	414912	63	0.90
FLT3	F° TGTTCACGCTGAATATAAGAAGGAA<br>R° GGAGCAGGAAGCCTGACTTG	100515445	70	0.92
FcεRIα	F° CAGGTGTCCTTGAATCCCCC<br>R° GGCATCTGTATTTGCCGCTG	100152827	74	1.07
BAFF /TNFSF13B	F° GGAGACGGTCCCCATCCT<br>R° AGCAGCTTCCCATCTTTGGA	100038026	69	1.09
IL10	F° GAGCCAACTGCAGCTTCCA<br>R° TCAGGACAAATAGCCCACTAGCTT	397106	65	1.01
IL21	F° AAATAGTCATCTGCCTGATGGTCAT<br>R° AGGCGATCTTGTCCTTGGAA	403123	76	1.01
***N* Lena** *viral RNA*	F° ATGGCCAGCCAGTCAATCAG<br>R° GGAACGTTCAGTTCCGGTGA	1494888	166	0.95
N FL13 *viral RNA*	F° GGGAATGGCCAGTCAGTCAA<br>R° ATCTTCAGCAGCTAGGGGGA	1494888	134	0.95

Supp Table 1: Primers used for RT-qPCR

a

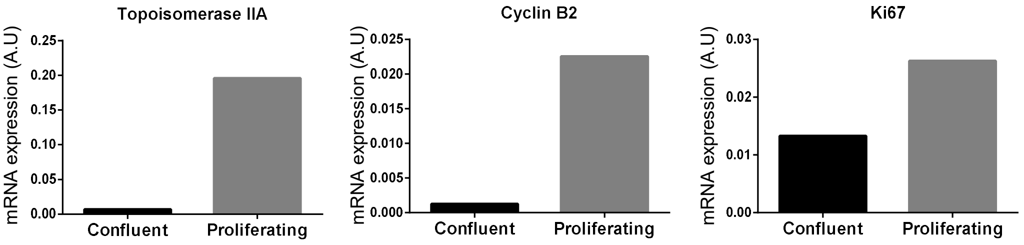

b

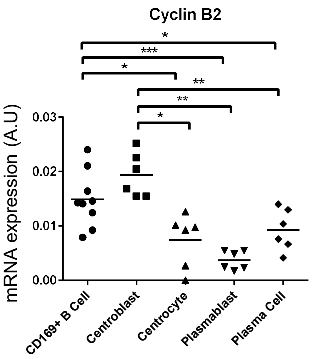

Supp Figure 1: Validation of proliferation measurement by RT-qPCR and lymph node B cells proliferation status.

(a) The porcine, trachea-epithelial cell line NPTR was harvested at confluence (confluent) or during exponential growth (proliferating). Transcriptional expressions of Topoisomerase IIA, Cyclin B2 and Ki67, 3 genes related to proliferation were measured by RT-qPCR. **(b)** Cyclin B2 RT-qPCR expression in LN B cell sorted populations. Arbitrary unit: A.U. represents 2-ΔCt.

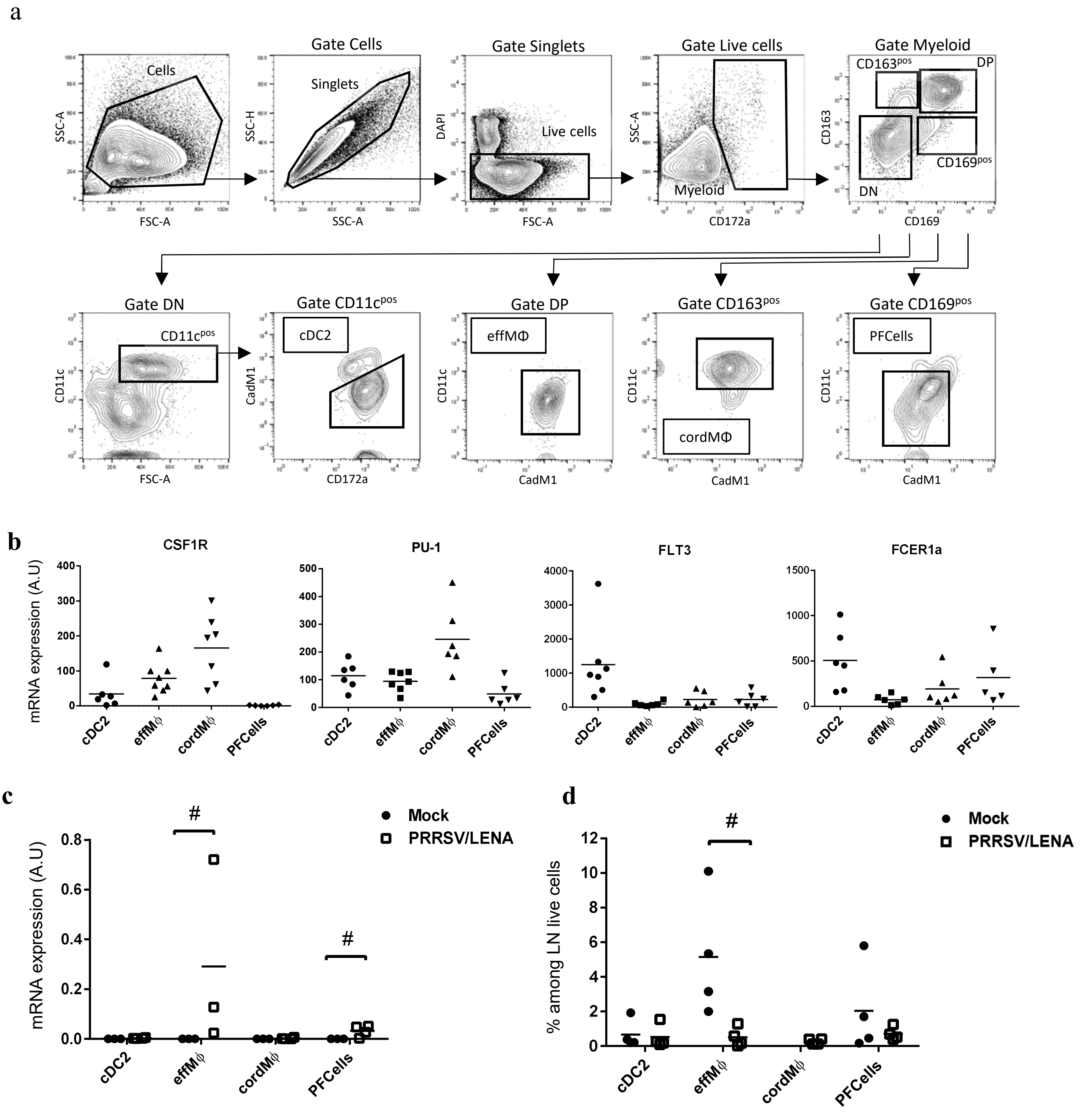

Supp Figure 2: Lena PRRSV infection of tracheobronchial lymph nodes MΦ and DC.

(a). The gating of effMΦ, cordMΦ, type 2 conventional dendritic cells (cDC2, defined as CD163^{neg}/CD169^{neg}/CD11c^{pos}/CD172a^{pos}/CadM1^{pos}), and perifollicular cells (PFCell) which represent a mix of PFMΦ and CD169^{pos} B cells. DN: Double negative cells (CD169^{-}/CD163^{-}); DP: Double positive cells (CD169^{+}/CD163^{+}). **(b).** Sorted cells were tested by RT-qPCR for Macrophagic and DC specific gene expressions. **(c).** Detection of viral RNA were performed on sorted cells by RNA extraction and RT-qPCR. **(d).** Evolution of population upon infection were analyzed as a percentage of each population among the total LN live cells extracted from flow cytometry data in (c). n=4. Arbitrary unit: A.U. represents $2^{-\Delta Ct}$, using RPS24 as reference gene.
